# Barriers to using eHealth/mHealth platforms and perceived beneficial eHealth/mHealth platform features among informal carers of persons living with dementia: a qualitative study

**DOI:** 10.1186/s12877-023-04628-0

**Published:** 2024-01-06

**Authors:** Ellaisha Samari, Qi Yuan, YunJue Zhang, Anitha Jeyagurunathan, Mythily Subramaniam

**Affiliations:** Institute of Mental Health, Research Division, 10 Buangkok View, Singapore, 539747 Singapore

**Keywords:** Carer, Dementia, eHealth, mHealth, Technology, Qualitative

## Abstract

**Background:**

New technologies have brought about a new age of technology-enabled aids that can equip informal carers with the relevant resources for better care. These include but are not limited to facilitating access to healthcare providers, knowledge of caring for persons living with dementia, and sources of support for carers’ well-being. This qualitative study explores barriers to using eHealth/mHealth platforms and perceived beneficial eHealth/mHealth platform features among informal carers of persons living with dementia.

**Methods:**

An exploratory qualitative study design was employed. Semi-structured interviews were conducted among 29 informal carers of persons living with dementia in Singapore recruited via convenience and snowball sampling. The interviews were audio-recorded and transcribed verbatim. Thematic analysis was used to analyse the data.

**Results:**

The participants in this study identified several barriers to using eHealth/mHealth platforms, including personal preference, apprehension, poor user experience and lack of skills. On the other hand, knowledge of dementia, caring for persons living with dementia and self-care, a list of resources, social support, location monitoring and alert systems, and the ability to manage appointments and transactions were valuable features for eHealth/mHealth platforms.

**Conclusions:**

Despite the underutilisation of eHealth/mHealth platforms, carers expressed a keen interest in using them if they are functional and capable of reducing their care burden. The findings from this study can contribute to developing content and features for eHealth/mHealth interventions aimed at lightening carers’ burden in their day-to-day caring routine.

**Supplementary Information:**

The online version contains supplementary material available at 10.1186/s12877-023-04628-0.

## Introduction

Dementia is a syndrome characterised by significant impairment in cognitive functioning, affecting memory, thinking, behaviour, and daily functioning [1, 2]. Worldwide, more than 55 million people live with dementia, and this figure is projected to increase to 78 million by 2030 due to the world’s ageing population [2, 3]. Consequently, the cost of dementia will increase [2, 4], and more people will have to take on the role of an informal carer, which is often reported to be highly demanding [5].

Informal carers, such as relatives and friends, generally provide the majority of care to meet the basic needs of persons living with dementia in the community [6]. Informal caring is often associated with experiencing significant physical, psychological, emotional and financial burdens [5]. In addition to managing and dealing with challenging symptoms of dementia, such as impairment in daily living activities and psychological and behavioural disturbances [5, 7], carers often struggle with role conflicts, such as juggling work and caring [8]. Furthermore, carers often engage in intensive and lengthy care [9]. Under these circumstances, caring for persons living with dementia is often associated with burnout, depression, anxiety, and poorer health [6, 10–12]. Nonetheless, some studies highlight carers’ experience of positive outcomes from caring for persons living with dementia, such as experiencing feelings of gratitude, a sense of mastery, personal growth and being more resilient [13–15].

New technologies have brought about a new age of technology-enabled aids that can enhance healthcare services and information delivery. These are often referred to as eHealth and mHealth. eHealth refers to "health services and information delivered and enhanced through the internet and related technology" [16], and mHealth is defined as “medical and public health practice supported by mobile devices, such as mobile phones, patient monitoring devices, personal digital assistants, and other wireless devices” [17]. Mobile technology in healthcare can be advantageous, as these devices are highly personal, easily accessible, and cost-effective [18, 19]. Furthermore, mobile technologies have the potential to enhance the overall caring experience. They offer easy access to relevant resources and information related to caring [20, 21], along with tools to support care management for persons living with dementia [22, 23].

Sriram et al. [22] conducted a systematic review to assess outcomes and informal carers’ experience with assistive technology (AT) use in dementia care at home. In their review, they identified AT as being mainly used for safety and security purposes (e.g., tracking devices, safety alarms, and fall detectors), supporting memory and orientation of persons living with dementia (e.g., memory games and cognitive stimulation exercises), as well as for social interaction and leisure activities (e.g., robotic therapy ‘seal’ and simple mobile phone). In general, AT was perceived as valuable and removing worries and burdens. In another systematic review, Brown et al. [24] explored how smartphone technologies have been used to support caring for persons living with Alzheimer’s Disease (AD). They had identified 13 apps to be relevant, of which eight focused more on the management of care towards persons living with AD (e.g., management of appointments and medication and hospice support). The authors noted that most of these apps had limited features and could not meet the complex needs of carers. Nonetheless, it is worth noting that mobile app-based interventions have shown some merit in reducing the burdens and improving the health outcomes of carers, including a decrease in carers’ anxiety and distress, depression symptoms and an increase in their sense of competence [20, 25].

In Singapore, the prevalence of dementia among residents aged 60 years and above is approximately 10% [26], and informal carers are reported to spend considerable time on caring [27]. In terms of sources of healthcare information, management of care and delivery of healthcare services, there are some websites (e.g., ‘HealthHub’ [28], ‘Agency for Integrated Care (AIC)’ [29] and ‘Jaga Me’ [30]) and mobile applications (e.g., ‘CARA’ [31]) supporting carers of persons living with dementia that are developed in Singapore. Other key features of the mobile applications include locating and notification of missing persons living with dementia, involving multiple family members in caring through a connected care circle feature, providing rewards to users in the form of discounts and privileges from partners (CARA) [31], and allowing users to view health and wellness information, access health records, and perform transactions for health services for themselves and their relatives (HealthHub) [28].

Understanding carers’ experiences with technology in supporting their caring journey is crucial for evaluating the relevance and utility of this medium in enhancing support and reducing the burden on carers. While existing research on mHealth [32–35] and eHealth interventions [36–38] has predominantly focused on Western contexts, it is important to consider the specific needs and experiences of carers in Asian countries. However, it is noteworthy that Singapore, being an Asian country, boasts well-developed technological systems that may bear similarities to those in developed or Western countries. Despite this, there remains a scarcity of research conducted in Asian countries to explore the attitudes and experiences of dementia carers in utilising eHealth/mHealth to support their caring efforts. Carers in Asian countries may have distinct needs and experiences compared to their Western counterparts. Moreover, the high prevalence of smartphone usage among Singapore residents further underscores the relevance and significance of exploring this topic in Singapore. With rates exceeding 95% among individuals aged 15–49, 88% among those aged 50–59, and 56% among individuals aged 60 and above [39], there is a clear indication of the potential for technology-enabled interventions to impact carers’ lives positively.

Therefore, conducting research in this area can inform our understanding of the experiences and effectiveness of eHealth/mHealth in supporting carers of individuals living with dementia in an Asian country. It can also provide insights for developing technology-enabled aids that better address the unique needs of carers in this population. To achieve these aims, the present study utilised a qualitative approach to identify barriers to using eHealth/mHealth platforms and gather suggestions for beneficial features of these platforms that can aid carers of persons living with dementia. Additionally, this study seeks to construct a conceptual framework that depicts the relationship between barriers to eHealth/mHealth use, beneficial features of eHealth/mHealth platforms, and the use of eHealth/mHealth platforms.

## Materials and methods

### Study design

This study employed an exploratory qualitative study design. Semi-structured interviews were conducted to gather the perspectives of carers of persons living with dementia regarding the use of eHealth and mHealth in their daily caring activities. The reporting of this study adheres to the Consolidated Criteria for Reporting Qualitative Research (COREQ) checklist [40], ensuring comprehensive and transparent reporting of qualitative data.

### Sample

Participants were informal carers of persons living with dementia in Singapore who were recruited between April 2019 and December 2020 via convenience and snowball sampling if they met the following inclusion criteria: (1) Singapore residents (Singapore Citizens and Permanent Residents; (2) aged 21 years and above; (3) key informal carer for a person living with dementia; and (4) able to speak in English, Mandarin, Malay or Tamil. Participants were recruited via referrals from clinicians treating their relatives (persons living with dementia) during their outpatient visits at the outpatient and satellite clinics of the Institute of Mental Health (a tertiary psychiatric hospital) or the geriatric clinic of a local Hospital. Study flyers were also disseminated to study participants for snowball recruitment of other carers. Participants also consisted of those who had participated in prior studies and had given permission to be contacted for future studies. Exclusion criteria included (1) carers who did not visit their relatives (persons living with dementia) every week, (2) carers whose relatives were institutionalised at the point of recruitment, and (3) carers who had difficulties understanding the informed consent document. The last criterion was implemented to ensure that participating carers possess adequate language and comprehension abilities, enabling them to provide informed consent and actively engage in the research study.

After discussing the research’s purpose, process, and data management, all participants provided written consent before the interviews. Consent-taking procedures were conducted by study team members (ES, QY, YZ, AJ) and not by referring clinicians to prevent coercion. A total of 43 people were approached via telephone calls. Of those approached, 29 participated and completed the interview. Those who did not participate were uncontactable (from the list of participants recruited from prior studies and permitted to be contacted for future studies) or refused to participate. While not all individuals provided reasons for their refusal, some mentioned they were not keen, busy, or uncomfortable participating in the interview. There was no established relationship between participants and study team members before study commencement.

### Data collection

Data were collected via semi-structured in-depth interviews conducted either face-to-face at a private place (e.g., private space in a public area or home) preferred by the carers (n = 23) or through online videoconferencing using the Zoom platform (n = 6) with 29 participants. Only participants and researchers were present for all interviews except for one where a family member also sat in with the participant. The online videoconferencing mode was extended to participants in light of the COVID-19 pandemic restrictions. Face-to-face interviews were conducted at public spaces or participants’ homes to ensure convenience. Most participants were bilingual in English and their mother tongue (i.e., Mandarin, Malay or Tamil), and they were allowed to have the interview conducted in either of these languages. Interviews were conducted primarily in English by male (QY, PhD) and female (ES, BA; YZ, MPH; AJ, MPhil) researchers trained in qualitative research and had prior experience conducting interviews. English was the most preferred language among participants. Hence, the interviews were predominantly conducted in English. However, some interviews were conducted in Mandarin (by QY and YZ) to accommodate participants who were more comfortable conversing in their mother tongue. The data used in this study originated from a qualitative study that aimed to explore carers’ experiences in providing care for persons living with dementia [15]. Additionally, participants were asked to share their opinions on the use of technology and their personal experiences with it, if applicable.

The interview questions were derived and refined through a literature review. Subsequently, to ensure their comprehensibility, an informal assessment was conducted with a few older adults who were family members of our colleagues. The primary objective of this evaluation was to verify whether the interview questions were easily understandable for older adult carers. Based on the feedback received from the older adults, no modifications were made to the interview guide, as they indicated clear comprehension of the questions. The interview questions included: 1) Do you have any experience using eHealth/mHealth programs to help you take care of [the person living with dementia]? 2) What is your opinion about these programs? 3) Are there any additional functions/features you would like to see in the programs? 4) Is there a reason why you are not keen to use such programs? 5) If we can solve [your concerns], would you use it?

Data collection ceased upon reaching saturation, which was the point at which no further new information or distinct themes were observed from the data. Saturation was determined through a thorough analysis of multiple interviews and careful examination of the data. This approach ensured that the qualitative data was rich and varied, allowing for a comprehensive exploration of the research topic. Interviews were audio-recorded, transcribed verbatim, and checked for accuracy by study team members. Transcripts in Mandarin were translated into English by QY and YZ, who are proficient in both languages. Interviews lasted 60 min on average, ranging between 33 min and 1 h and 35 min. No repeat interviews were carried out.

### Data analysis

Transcripts were given codes in the form of P01, where P = participant and the numerical number represents the recruitment order (e.g., P01, P02, P03). Nvivo11, a qualitative data analysis software, was utilised to manage and analyse all the transcripts.

Four study team members (ES, QY, YZ, and AJ) analysed the data using thematic analysis as informed by Braun and Clarke [41]. The authors employed an inductive-deductive approach during the data analysis process. Initially, using the inductive coding method, each team member independently read one separate transcript and coded meaningful data units. Through team discussions, these initial codes were generated into higher-order concepts, forming the initial coding framework [42].

In the deductive phase, the authors utilised pre-existing concepts, specifically the barriers to using eHealth/mHealth platforms in caring and beneficial features of eHealth/mHealth platforms, to categorise the emerging themes within the coding framework. Three randomly selected transcripts were then individually coded by coders ES, QY, YZ, and AJ using the coding framework to validate and refine the framework. Extensive comparisons and discussions of their coding were conducted, resulting in adjustments and improvements to the framework.

Subsequently, all 29 transcripts were distributed among coders ES, QY, and YZ for independent coding using the refined coding framework. During this process, new codes emerged, prompting the authors to carefully examine and discuss their relevance and potential significance. These new codes were then compared to the existing coding framework, leading to further refinement and expansion to accommodate the newly identified codes. By incorporating these newly identified codes into the analysis, the authors ensured a comprehensive approach that enriched the interpretation of the data and contributed to the overall study findings. The current analysis for this study focused on attitudes and experiences with technology in caring for persons living with dementia. Specifically, key themes were generated from participants’ narratives on the barriers to and suggestions for using technology to care for persons with dementia.

## Results

Twenty-nine dementia carers, comprising six males and 23 females, completed the interview. They had a mean age of 56.3 ± 6.5 years (range: 46–72 years). The majority of participants were Chinese (n = 26) and were daughters caring for their mothers (n = 20). Fifteen dementia carers had helpers to assist them in caring for persons living with dementia.

Most participants reported having utilised eHealth/mHealth at some point in their caring journey; however, the frequency and extent of use varied, with most responses suggesting its underutilisation. Participants spoke of accessing health-related websites and using mobile applications for caring purposes. The primary uses of such platforms included: 1) gathering information on topics including dementia, caring strategies, as well as caring support and resources, 2) managing patients’ health information and transactions (e.g., accessing medical records, booking appointments, paying bills and refilling medications), 3) consulting medical professionals, 4) caring for the patient (e.g., fall detection, tracker and automated reminders for persons living with dementia) and 5) seeking social support (e.g., online support group). The mobile applications used by some participants to support their caring were HealthHub and Dementia Friends [43] (which has been replaced by the CARA app). In addition, one participant mentioned having used an app that was developed as part of a study but was later discontinued due to the high cost of maintenance for the app.

In all, two broad themes and nine subthemes emerged from the data. Table 1 displays the overview of themes and subthemes.

**Table 1:** Overview of Themes and Subthemes

Themes	Subthemes
Barriers to the use of eHealth/mHealth platforms in caring	Personal preference and apprehension towards eHealth/mHealth
	Lack of knowledge, skills and bandwidth
	Poor user experience of mHealth
Perceived beneficial features of eHealth/mHealth platforms	Knowledge related to dementia, caring and self-care
	List of supporting resources and helplines
	Social and emotional support from support groups
	Usability of eHealth/mHealth platforms
	Location monitoring and alert systems
	Management of medical appointments and transactions

### Barriers to the use of eHealth/mHealth platforms in caring

While some participants reported experiencing greater convenience when using technology to aid them in their caring journey, others were not keen on using it for varying reasons. In addition, some carers thought that technology-enabled aids tend to appeal only to those who were technology savvy.

#### Personal preference and apprehension towards eHealth/mHealth

Many participants preferred face-to-face interactions over technological mediums when interacting with another person. They believed telemedicine and interactions with health professionals over technology, such as teleconsultation, often lacked the personal touch they desired. One participant (P24/F/66) captured this sentiment by stating, “*Yeah, it’s always personal, face-to-face consultation and talk, so much easier because can see the person, physically see them. But over internet, I think it takes a while to adjust*.” This opinion reflected the feelings of many participants who believed that consultations are deeply personal. Thus, they felt it would be more appropriate and familiar to them to have them conducted in person.

Some participants preferred to seek information and support from their support groups, compared to relying on apps or technological resources. One participant (P25/F/47) highlighted this by stating, “*For me, I don’t rely on apps, I rely on my own support group, that’s where I get all the stuff I need… to support my mom’s …me and my mom’s journey*.” This perspective underscores the value of personal connections and the support gained through interpersonal relationships. While some participants found assistance through support groups, others mentioned their preference for having hard copies of helpful information, citing enhanced readability and convenient access as reasons for their choice.

Some participants expressed apprehension towards using technology, primarily due to the uncertainty surrounding the authenticity of online content. Specifically, they mentioned the difficulty distinguishing between authentic and inauthentic content, leading them to prefer receiving information from trusted sources, such as individuals with first-hand experience.

‘Some [online content] is genuine, some is not genuine, and so much so to a stage where I come in, I thought, I cannot believe everything. So, but, if it comes from a person who themselves are taking care of their loved ones who got dementia, which is my own lecturer, of course I’ll, I’ll go with him’ – P01/M/55.

#### Lack of knowledge, digital skills, and bandwidth

Many participants cited various reasons for their limited use of technology. These included a lack of knowledge on how to use it and uncertainty about where to find the necessary resources. One participant (P28/F/72) said, “*First thing, I don’t know how to use. Second, (I) also don’t know where to get all these things.*” In addition, participants mentioned bandwidth constraints and a sense of unease and unfamiliarity with using technology, which made it challenging for them to navigate and utilise it effectively. For example, one participant (P06/M/68) explained, “*I’m very, very low-tech, you know, because of my age… I’m articulate in a lot of ways, but I think half of my brain is not skewed to such high technology, and that’s my feeling, I think*.”

Another participant mentioned that learning something new is taxing for carers because they are already overwhelmed with their responsibilities. Nonetheless, he acknowledged that it is helpful for those who value its utility and would like to use it.

**‘**I think when some people are walking this journey, mentally they are already overburdened, mentally they are very closed already. You just go and tell them one more thing they cannot absorb. But, but it’ll be useful to always have, it’ll be useful to always have some of this in case there are some people who like to, who want to use it.’ – P20/M/64.

#### Poor user experience of mHealth

A few participants reported poor user experience when using technology to aid them in caring. These stemmed from challenges regarding the incompatibility of their devices with the mobile application to poor performance of the app’s functions (e.g., inaccurate tracking location and insensitivity of fall detection). In addition, some reported finding it cumbersome to navigate the apps and that they were inadequate in providing the information they sought.

‘Some of the problems I encountered, for example, I think although it’s only a once off, or maybe just twice out of the whole three months, the tracker did not quite really show the location of where my mom really is…there was once that it’s very shocking when I, when I turn on the app to see where my mom is, it shows that she, the location shows that she’s somewhere in South China Sea so I was like what? I mean, I know that she was going to my sister’s place, but somehow that, that situation, it, it shows that she was at South China Sea.’ – P10/F/56.

### Perceived beneficial features of eHealth/mHealth platforms

Most participants voiced their receptivity and interest in using eHealth/mHealth platforms designed for carers if they included helpful features that could assist them in caring for persons living with dementia or enhance their well-being. Some participants who had experience using eHealth/mHealth platforms also provided suggestions for improving these platforms.

#### Knowledge related to dementia, caring and self-care

Among the participants, a commonly voiced ideal component of eHealth/mHealth platforms for carers of persons living with dementia was providing information and knowledge related to improving dementia literacy, coping, self-care and ways to engage persons living with dementia. In relation to dementia literacy, topics suggested included its aetiology, prognosis, medication, as well as recognition and management of symptoms. Some participants had also spoken of wanting content curated for the different stages of dementia.

‘Um, maybe it’s the recreation for dementia patients, what is suitable or what is not suitable or some ways to keep them occupied… Yeah, because how do you prevent them from deteriorating? And then I tell her to exercise, she doesn’t exercise, so what to do?’ – P21/F/60.

‘Maybe, like for her case, dementia, there is different stages, right? So like, what to expect from stage 1, stage 2? How do we know? Or which stage she is in? or what are the how will she deteriorate in the future?’ – P13/F/50.

Aside from wanting to be equipped with coping and self-care strategies, such as dealing with difficult emotions and promoting good health, some participants noted that highlighting the importance of self-care would also benefit users.

‘Constantly feed this type of information – how to like self-care… You got to insist, you got to stress a bit you need to self-care or what are the self-care that you can do that kind of thing, that’s one thing. Second thing, the typical thing is the information like – you don’t feel guilty when you behave this and because of taking… when you struggle with them these sort of information yah.’ – P08/M/56.

#### List of supporting resources and helplines

Participants remarked that they often needed support in managing care for persons living with dementia; thus, having a list of resources that could direct them to the relevant services, suppliers of products needed (e.g., care equipment and accessories, toys, books, etc.) and helplines would be highly useful to them. While the commonly mentioned services that carers needed were focused on care for persons living with dementia, such as transportation (e.g., ambulance and wheelchair-friendly transport), care at centres (e.g., daycare, respite care and interim care), care at home (e.g., helper (relief, part-time or full-time), house call doctors, nurses) there were also some mentions of including resources that focused on carers’ wellbeing such as hotlines for counselling services.

‘Due to my contact, I know of a shop that sells elderly-friendly toys or dementia-friendly accessories. For example, they will have a pet cat; a cat feels like a real cat but have to sing songs, but it’s a mechanic toy for people with dementia or even babies. Yeah…so it will be good to list that as part of the app, so people will know that if I want to get resources like this, where can I get it, or maybe puzzles and stuff? Other than that, maybe medical equipment, that could be part of it.’ – P25/F/47.

Additionally, some participants suggested that carers could benefit from information on the relevant courses and workshops to support them in their caring journey and promote their overall well-being. One participant (P03/F/56) highlighted this by saying, “*Um…maybe some courses also…courses where you can attend…yah. I know there is a course on mental health. Yeah, so some courses related for you to attend, which I think that will be helpful also.*”

In particular, a few carers who had helpers highlighted the need for a feature or resource list that facilitates connections with available helpers on a relief, part-time or full-time basis or respite care. This would be especially helpful when they or their helpers are unavailable to care for the person living with dementia. As one carer (P23/F/56) expressed, “*Let’s say …if helper want to go off can send someone over for one day…for a few hours…maybe…don’t know whether they provide this kind of service…have this type of service.*”

#### Social and emotional support from support groups

A few participants suggested features that could support users socially and emotionally. These features should ideally connect them to a support group or function as an outlet (e.g., a forum) for them to air their thoughts. Being connected to a support group was especially useful for carers as they could talk to someone who could relate to their experiences or learn from those who share similar experiences.

‘…if you actually have not walked the journey, you cannot sit down and try to write the info because you do not know what is it. So, so, I actually think if people are writing this, it’ll be people who are, people who are writing and doing this will be people who are actually handling, handling the patients or the caregivers; they must be the ones handling it otherwise I doubt, I doubt the usefulness of the information…In this case, the support groups, support groups may be, may be a better [option]… for us. Some of these can be tips and all that.’ – P20/M/64.

‘I don’t know what to expect from an application. I guess you can put it in a forum when you are frustrated, and you can just write away whatever you want to write, and no one is going to judge you about it’ – P29/F/52.

#### Usability of eHealth/mHealth platforms

According to some participants, being comprehensive, relevant and easy to use were features that would enhance the usability of eHealth/mHealth platforms. Specifically, some mentioned their preference for content that is ‘*easily accessible and digestible*’ (P07/F/58), ‘*not too complicated to use*’ (P19/F/52), ‘*not too difficult to understand*’ (P19/F/52), ‘*not too long*’ (P08/F/53), and an app that is ‘*integrated*’ (P26/F/49).

A few participants also suggested having educational videos as an alternative to delivering caring information. For one participant, a video clip would be handy to show her helper, who may not be well-versed in English (to understand her instructions fully), how to perform the necessary task:

“Let’s say you want to clean her, her tube feeding, you know what’s the way you know. Of course, IV [Intravenous Tubes], I dare not to touch, so I’ll tell her [helper], you do this; this is the right way. Then,(if) there’s a video, then the helper also can see you see… Demos to show things so that it’s easier to explain to a less English-speaking maid, you know, yeah.” – P05/F/62.

#### Management of medical appointments and transactions

Some participants spoke of wanting the ability to manage medical appointments and transactions, such as purchasing and paying for medications, through eHealth/mHealth platforms. They suggested the possibility of an application that would enable carers to order medications and conveniently collect them from the hospital. As one participant (P11/F/54) mentioned, “*Maybe there is an application that you can order medication from, and then you just go to the hospital and then collect. Yeah, that’s a good thing also because sometimes the medication can be a long time.”* This feature would allow carers to avoid the inconvenience of visiting medical institutions and enduring long waiting times for prescription collection.

Furthermore, one carer (P09/F/53) highlighted her grievances in having to call in to arrange for appointments, stating, “*What would help is appointments, making appointments, because very hard for us to call appointment hotlines, not nice, it’s a headache. My mom had a dental at XXY [local hospital], I had to make multiple calls and, yea, to just settle. It took me like three weeks; you know to call various parties and all that.”*

#### Location monitoring and alert systems

Some participants highlighted the importance of incorporating location monitoring features to help locate lost individuals, automated prompts or reminders (e.g., for taking medication), and medical alert systems (e.g., an emergency button) within eHealth/mHealth platforms. In addition to conventional methods of tracking individuals through wearable devices and location tracking, one participant suggested an additional feature: allowing users to drop a pin at the location where a wandering or lost person living with dementia is found, accompanied by descriptions of the person. This functionality would trigger an alert and guide carers directly to the person’s location.

‘That [tracking feature] would be a useful thing to have. So that in the event … that means…if people are … I mean not only for me as a caregiver… people who are able to find lost …the people who are lost due to their dementia they can actually bring them to such location. That is one feature I was thinking of.’ – P15/F/52.

### Conceptual framework

**Fig. 1:**
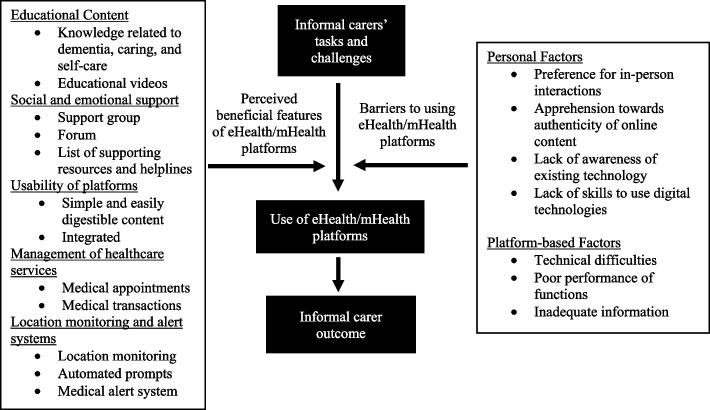
A conceptual framework for the use of eHealth/mHealth platforms in caring

We have synthesised our study findings into a conceptual framework, as illustrated in Fig. 1. This model depicts the relationship between barriers to eHealth/mHealth adoption, beneficial features of eHealth/mHealth platforms, and the use of eHealth/mHealth platforms.

## Discussion

While technology is evolving rapidly, its uptake moves more slowly. However, existing technology can be maximised to strengthen the resources and address the concerns and needs of informal carers of persons living with dementia in their day-to-day lives. This can be done through consumer devices (e.g., smartphones and laptops). This qualitative study adds to the literature on barriers to using eHealth/mHealth and perceived beneficial features of eHealth/mHealth platforms among carers of persons living with dementia in an Asian setting. Data from this study can provide directions for developing eHealth/mHealth interventions aimed at addressing the needs of carers of persons living with dementia and supporting their well-being.

Findings from this study suggest that personal factors (e.g., preference for in-person interactions, apprehension towards the authenticity of online content and poor digital literacy) and platform-based factors (e.g., technical difficulties, the poor performance of functions and inadequate information) impede the use of eHealth/mHealth as a supporting tool for carers. Notably, while platform-based factors can be improved, personal factors may or may not be modifiable. Specifically, some participants spoke against telemedicine. Telemedicine is an expanding model of care for patients and their carers [44]. Conducted via synchronous videoconferencing, telemedicine is advantageous in providing care for older adults with dementia as it allows clinicians a glimpse into the patient’s environment at home [45], in addition to increasing access to care, especially for those who find it difficult to leave their homes, and reducing costs. However, despite its advantages, some participants felt telemedicine reduces that ‘personal touch’. Similarly, the challenge of telemedicine in establishing rapport and personal connections resonates with other studies among physicians [46]. In this vein, for those concerned with losing ‘personal touch’, telemedicine could complement in-person consultations with physicians with whom they have already established rapport and assessments of the home environment or situations in which mobility is affected.

Consistent with current literature, participants’ lack of awareness of existing technology and poor digital skills [47] were barriers to using technology in supporting their caring tasks and well-being. Efforts can be made to address this gap and raise awareness of existing and relevant technology or eHealth/mHealth platforms, mainly through medical professionals whom they are often in contact with during medical visits, so that informal carers can maximise the benefits of improved carer outcomes that eHealth/mHealth platforms can potentially offer. However, according to studies of older adults and carers, while medical professionals are deemed the most trustworthy source of technology recommendations, medical professionals reported that they are not up to date with the latest consumer technologies [48]. In this light, as medical professionals appear to play a crucial role in educating carers in finding credible and reliable sources, in addition to recommending established platforms, the first step could be to equip medical professionals with knowledge and skills on relevant technologies before having them recommend it to carers. Having medical professionals recommend credible and reliable sources could also ease carers’ worries towards the authenticity of the content.

Despite these barriers, there remains great potential for using eHealth/mHealth platforms to support informal carers in their day-to-day tasks and well-being. Informal carers raised several key features to be included in eHealth/mHealth platforms that can support them as carers. Firstly, they voiced the need for more information, specifically about dementia, caring for persons living with dementia, and improving carers’ well-being through coping strategies and self-care. In particular, this study highlighted that informal carers proposed content curated to the different stages of dementia. This need for more information is echoed in prior studies [49, 50] and underlines the importance of having it on eHealth/mHealth platforms for carers. A considerable benefit of eHealth/mHealth platforms is the ability to connect users to vast amounts of information in various modes, such as through video recordings. Providing informal carers with information they can relate to can lessen their level of fatigue [51] and alleviate carer depression and burden [52].

Secondly, informal carers suggested features allowing users to connect to other carers, such as forums or support groups, to receive social and emotional support from others with similar experiences. This is an important feature considering the negative psychological, social and financial consequences associated with caring in the dementia context [53, 54]. However, functions that target the emotional needs of informal carers, such as forums to share experiences and evaluations for their well-being, are less common than features that provide information related to caring tasks or educational information within eHealth/mHealth platforms [21, 55]. eHealth/mHealth platforms which promote social relations and connections with other informal carers, can enable users to obtain information from others with similar experiences or to share their experiences and receive validation without having to attend in-person sessions that may be difficult for those with transportation issues or difficulties in finding respite care. Lee reviewed the effectiveness of technology-based support groups in reducing the care burden for carers of persons living with dementia [56]. Among the five studies reviewed, three utilised Internet support, one used telephone support and one used both Internet and telephone support. All five studies showed that technology-based social support groups reduced the care burden among carers of persons living with dementia. In addition, in one of the studies reviewed [57], the authors found that video conferencing interventions were more helpful and effective than chat group interventions as they allowed face-to-face support.

Enabling video conferencing on eHealth/mHealth platforms for social support groups or between users could thus be considered for developing future eHealth/mHealth platforms to increase the effectiveness of social support in reducing care burden. Similar to findings from other studies, the acceptability of eHealth/mHealth platforms is highly dependent on a simple user interface and features that are easy to use or require low effort [58, 59, 60]. Other studies have also suggested appropriate font types and sizes in designing user-friendly platforms [60] or those which are easy to install and reliable [61]. Notably, it appears to be particularly important for eHealth/mHealth platforms to be simple for older adults, bearing in mind normal age-related cognitive decline, which has been found to impede the use of technology among other older adults [62]. Likewise, participants in the current sample had alluded to normal age-related cognitive decline as a reason for their inability to learn new technologies; thus, having features that are easy to use or a simple interface might encourage the use of eHealth/mHealth platforms amongst this population.

Many carers cannot attend in-person educational or support sessions due to the demands of caring, making internet-based services a seemingly more feasible option. Furthermore, akin to the current study findings, informal carers are generally receptive to eHealth/mHealth interventions that can meet their needs and reduce carer burden, such as providing relief to their geographical, time, financial and spatial constraints [63, 64]. Notably, data from this study were partly gathered before the COVID-19 pandemic. Healthcare delivery, including digital care for dementia patients and their family carers, has changed significantly [65]. The implementation of social distancing measures  has prompted healthcare providers to pivot towards telemedicine to deliver care to patients living with dementia. Concurrently, there was an increase in online support groups for family carers to resolve the challenge of social isolation. Furthermore, there have been reports of increased utilisation of mHealth applications during the COVID-19 pandemic, as enforced restrictions have necessitated innovative approaches to healthcare delivery [66, 67]. Therefore, researchers need to continue exploring new and more effective ways to provide carers with the help and support they need in the context of mHealth and the likely increase in familiarity with digital technology due to changes ushered by the COVID-19 pandemic.

### Study limitations

Most participants were unaware of existing eHealth/mHealth platforms, thus limiting data gathered on user experience and recommendations for existing eHealth/mHealth platforms. This highlights the need for additional research to evaluate existing eHealth/mHealth interventions for informal carers or persons living with dementia.

## Conclusion

While the study indicated the relative lack of awareness and underutilisation of eHealth/mHealth platforms in caring, carers showed interest towards them if they are functional and can reduce the caring burden. Addressing barriers to its use and incorporating perceived beneficial features of eHealth/mHealth platforms for carers can hopefully lead to maximising the utility and benefits that eHealth/mHealth platforms can provide for carers of persons living with dementia. Overall, results from this study can inform the development and implementation of eHealth/mHealth interventions aimed at lightening carers’ burden in their day-to-day caring routine or guide healthcare professionals and eHealth/mHealth platform developers in designing better platforms that are more effective and better accepted among older adult carers. Further research on the health and digital literacy of carers of persons living with dementia is also needed to complement and enhance the utility and applicability of the current study findings in developing acceptable eHealth/mHealth platforms for them. In addition, promoting such platforms is very much needed among the carer community. Future research could also evaluate existing or newly developed eHealth/mHealth platforms.

### Supplementary Information

**Additional file 1.** Consolidated criteria for reporting qualitative studies (COREQ): 32-item checklist.

## Data Availability

Data for this study are available upon reasonable request. The data request can be sent to The Institutional Research Review Committee, Institute of Mental Health, Singapore; Email address: imhresearch@imh.com.sg.

## Abbreviation

AD: Alzheimer's Disease
AT: Assistive technology
